# “To have children or not?” Between desire, responsibility, luck, and guilt: reproductive decision-making in individuals with neurofibromatosis type 1

**DOI:** 10.1186/s13023-025-04061-z

**Published:** 2025-10-21

**Authors:** Katarzyna Kowal, Jan Domaradzki

**Affiliations:** 1 Wladyslaw Bieganski Collegium Medicum, Jan Dlugosz University in Czestochowa, Aleja Armii Krajowej 13/15, Czestochowa, 42-200 Poland; 2 Department of Social Sciences and Humanities, Poznan University of Medical Sciences, Poznan, Poland

**Keywords:** Disease experience, Neurofibromatosis type 1, Rare disease, Genetic risk, Reproductive decision-making, Responsibility

## Abstract

**Background:**

Neurofibromatosis type 1 (NF1) is a rare autosomal dominant disorder with a 50% chance of being passed to offspring. Its hereditary nature presents individuals with complex reproductive dilemmas. This study explores the complexity of decision-making and reproductive choices faced by people with NF1 regarding parenthood.

**Results:**

Genetic risk is a key factor shaping reproductive decisions. For some individuals, the decision not to have children is seen as a protective and morally responsible practice, aiming to spare potential offspring from the stigma and isolation they themselves experienced. Some women were also concerned for their own physical and emotional health, especially in relation to pregnancy and caregiving. Medical professionals’ opinions significantly influence choices, sometimes outweighing personal desires for parenthood and shaping perceptions of reproductive responsibility. Parents who were unaware of their diagnosis at the time of conception express guilt and regret. Despite the risks, many still wish to have children but struggle with the fear of passing on the NF1 mutation and potential difficulties in bonding with a child who may also be affected. Individuals who realized procreative plans despite severe NF1 treat parenthood as an important element of their non-disease identity and a source of emotional strength.

**Conclusions:**

For individuals with NF1, reproductive decision-making is a complex dilemma, in which procreation anxiety intersects with hopes for parenthood, a sense of responsibility for the child’s future, and personal identity.

## Background

Neurofibromatosis type 1 (NF1) is a rare genetic disease with autosomal dominant inheritance, previously classified as phacomatoses, and today is included in the group of so-called RASopathies. RASopathies constitute a group of genetic diseases caused by mutations in genes regulating the RAS-MAPK signalling pathway. This pathway is responsible for controlling cell growth and division. The RAS oncogene plays a key role in it [1]. Therefore, RASopathies are characterized by an increased risk of developing cancers. Although this applies especially to benign tumours, which are a characteristic symptom of NF1 (neurofibromas), in NF1 there is also a predisposition to malignant tumours (e.g., breast cancer and leukaemia) [1]. Friedrich Daniel von Recklinghausen, a German pathologist, is considered to be the discoverer of NF1. In 1882, he published a paper in which he was the first to describe a new disease entity – neurofibromatosis [2].

NF1 is a relatively common autosomal dominant disease. Its estimated population prevalence worldwide is 1:1900 to 1:3500 [3]. NF1 is a disease with a high risk of passing on a gene mutation. A child of an individual with NF1 has a 50% risk of inheriting the pathogenic variant from a parent. If this happens, the child will develop NF1 with features that are more or less severe than in the affected parent. If the disease-causing variant is known in the family, prenatal testing is possible in the case of a high-risk pregnancy and genetic testing before implantation. Half of NF1 patients have a *de novo* mutation [4]. The estimated number of NF1 patients in Poland is about 11.000 [5].

The Riccardi NF1 severity scale has four levels. Level 1, minimal NF1: only a few characteristic features of the disease are present, without any threat to health. Level 2, mild NF1: there are clear symptoms that allow a clear diagnosis of the disease, but without a significant impact on health. Level 3, moderate NF1: there are significant health problems that worsen the patient’s condition, but which can be effectively controlled. Level 4, severe NF1: the presence of serious health problems that are difficult to control and manage, requiring complex treatment [6]. Studies conducted in Poland show that among individuals with NF1, patients with severity levels 2 and 3 predominated (50%). The average severity of NF1 symptoms was similar in the sporadic and familial groups, which suggests that the mode of disease onset (de novo mutation vs. inheritance) had no significant impact on the severity of the disease. Importantly, in the case of inheritance of the disease from parents, the average severity of NF1 in children was higher than the average severity of the parents’ disease. Moreover, children passed from NF1 severity level 1 to 2 earlier than their parents [7].

NF1 is a genetic disease with a highly variable clinical expression. Even within a single family, the symptoms of the disease can greatly differ in their severity, which is a significant diagnostic challenge. The symptoms of NF1 appear with age in almost 100% of patients [7]. Historically, the National Institutes of Health (NIH) Consensus Criteria from 1988 have long served as the gold standard for diagnosing NF1 [8]. These criteria required the presence of at least two out of seven clinical features: (1) six or more café-au-lait macules, with a diameter of ≥ 5 mm in prepubertal children and ≥ 15 mm in adolescents and adults, (2) two or more neurofibromas of any type or one plexiform neurofibroma, (3) freckles in the groin or armpit areas (so-called Crowe’s sign); (4) two or more Lisch nodules; (5) dysplasia of the sphenoid wing and/or cortical thinning of long bones, which may lead to fractures; (6) optic glioma; (7) a first-degree relative with NF1 (parent, sibling, or child) who meets the above criteria [8].

However, these criteria were updated in 2021 to reflect advancements in genetic diagnostics and clinical understanding of the disease [9]. Thus, NF1 can now be diagnosed either clinically or by identifying a pathogenic or likely pathogenic variant in the NF1 gene. The updated criteria include several important changes. The criterion of two or more Lisch nodules was expanded to include two or more choroidal abnormalities. The term optic nerve glioma was replaced with optic pathway glioma (OPG) to encompass tumors affecting not only the optic nerve but also other parts of the visual pathway. To improve diagnostic clarity, the criterion for bone abnormalities was refined to include characteristic features such as sphenoid bone dysplasia, anterolateral bowing of the tibia (tibial dysplasia), or pseudarthrosis of a long bone. Additionally, it was specified that at least one of the two pigmented skin findings (café-au-lait macules or freckling) must be bilateral. These changes aim to facilitate earlier and more accurate diagnosis of NF1, which is essential for effective disease management [9]. Despite these updates, many clinicians, including NF specialists, remain unaware of current NF1 guidelines, highlighting a gap in the dissemination and adoption of standardized recommendations [10].

Although recent years have seen some breakthroughs in the development of targeted therapy, particularly selumetinib, a MEK inhibitor that can shrink plexiform tumours, treatment for NF1 is primarily symptomatic [11]. NF1 is associated with a reduction in life expectancy of 8–15 years in both men and women, primarily as a consequence of malignancies and cardiovascular causes [3].

The issue of reproductive decisions in individuals with NF1 does not have many references in the world literature. Existing reports are consistent in that both the issue of sexuality and parenthood are challenges for individuals with neurofibromatosis. For both women and men with NF1, sexuality is a source of tension and difficulties. Their manifestations include problems with self-acceptance, but also with acceptance from sexual partners, the experience of rejection, stigmatizing judgments, conflicts, reluctance, emotional isolation of the sick individual, and finally breakdown of the relationship [12]. NF1 also imposes a burden on decisions regarding family life. Studies by Benjamin et al. [13] confirm that the experience of NF1 and knowledge about the risk of passing on the disease to offspring, in almost half of patients (45%) influences decisions regarding procreation. Most of these individuals did not resign from having children, but when considering the decision on parenthood, they reported the intention to perform prenatal diagnosis in a potential pregnancy. Only a few individuals in the study admitted that they would consider terminating the pregnancy if NF1 was confirmed in the foetus.

Reproductive decisions are associated with women experiencing serious ethical and emotional dilemmas, not only related to the inheritance of the disease by their offspring, but also to the risk of exacerbation of symptoms, especially the most visible ones. They are also accompanied by the awareness of a deep moral responsibility and the emotional burden of the decision to pass on a genetic disease to a child, who is potentially condemned to suffering. On the other hand, women with NF1 are aware of their own health risk, which sometimes has a stronger influence on the decision to refrain from procreation than the risk of heredity of the disease. In addition, there is the psychological burden associated with the decision not to have children and the resulting feeling of guilt in a situation when an individual with NF1 wants to have a child. The conflict here is between the desire for motherhood and the fear for the future of both the child and oneself [14].

The aim of this study is therefore to explore the reproductive dilemmas experienced by individuals with NF1. The specific objectives are: (1) to describe the complexity of reproductive decisions, and (2) to identify factors that determine reproductive choices and family planning in individuals with NF1.

## Material and methods

### Study design

This study is part of a larger research project led by the first author (KK), entitled *The experience of neurofibromatosis type 1 from the perspective of the sick individual and his family - a sociomedical study of a chronic disease*. The study aims to understand how NF1 is experienced by the sick individual and their family members in everyday life. The main research question concerned the meaning of the body altered by the disease and its impact on the self-concept as well as the personal and social identity of individuals with NF1.

The empirical basis of this text is part of the aforementioned study that focused on the way neurofibromatosis was experienced in the reproductive dimension. We refer to that part of the collected material in which the respondents reported their (un)fulfilled procreative plans, and also shared their emotions, fears, and moral dilemmas experienced in this area. Due to the lack of previous sociomedical studies on this topic, a qualitative strategy was adopted in the presented study. This approach enables a deep understanding of the impact of experiencing a rare genetic disease such as NF1 on reproductive decisions. Conducting in-depth interviews (IDI) with individuals with NF1 allowed us to capture: (1) the individual adaptive strategies of the respondents in relation to the risk of heredity of the disease; (2) the ways of interpreting the diagnosis in the context of the desire and right to have a child; (3) the mechanisms of making decisions regarding (not) having children, taking into account the ethical, social and health aspects.

The study was carried out in accordance with the guidelines of the Helsinki Declaration of 1964 (updated in 2000) and was approved by the Bioethics Committee of the Nicolaus Copernicus University in Torun, L. Rydygier Medical College in Bydgoszcz (KB resolution 617/2017).

The following study description was prepared based on the consolidated criteria for reporting qualitative research (COREQ) [15].

### Participants

The selection of the study group was determined by the criterion of expediency, according to which only individuals with a medically confirmed diagnosis of NF1 were invited to the study. Other inclusion criteria were: age over 18; willingness to participate in the study; providing informed consent; use of the Polish language; access to the Internet; and the ability to navigate it.

Before starting the study, it was necessary to find individuals who would act as informants (so-called gatekeepers) in reaching the respondents. They were two doctors caring for patients diagnosed with neurofibromatosis type 1, who helped to establish contact with potential respondents by sending them an invitation to the study. Recruitment for the study conducted in this way lasted continuously from April 1, 2020, to December 31, 2020. The patients who expressed their willingness to participate in the study contacted the study director themselves, who, after a short qualification (ensuring that a medical diagnosis of NF1 had been made), decided to include them in the study. The place of recruitment of the subjects was the Oncology Clinic - Fakomatoz at the Dr Antoni Jurasz University Hospital in Bydgoszcz.

The study included 93 individuals with neurofibromatosis type 1, including 69 women (74%) and 24 men (26%). The age of the study participants ranged from 18 to 64 years (mean 36.69 years, SD = 10.13). As for the age at medical diagnosis of NF1, the earliest time of NF1 diagnosis among the study participants was the 1st year of life, while the latest moment was 51 years of age. The average age of the respondents at the time of NF1 diagnosis was 17.55 years, SD = 12.18. The largest number of respondents were individuals who were not in a permanent relationship (marriage/partnership) and defined their marital status as single – 54 individuals (58% of respondents). In turn, 33 respondents were married (36%), five were divorced (5%), but separated (1%). 46 respondents declared having children (49%), while 47 were childless (51%). 30 respondents had one child, 14 had two children, and 2 respondents had three children. Of the 64 children of the respondents, 42 had a medical diagnosis of NF1 (65%).

None of the respondents had any relationship with the researcher, and there was no interaction between them before the research began. The researcher had no personal experiences or reasons for becoming involved in a research project on such a topic either. This information was also presented to the individuals invited to the study before it began. The researcher’s interest in the subject of the project was solely substantive.

### Data collection

The study was conducted between August 1, 2020, and April 18, 2021, by the first author of the manuscript (KK). Because of restrictions related to the COVID-19 pandemic, it was not possible to conduct the study in the originally planned form of direct meetings with the respondents. Therefore, in-depth interviews were conducted via telephone conversations and online meetings.

During the first contact with the respondents, the researcher presented the project topic, explained the research goals, its course, and the use of the collected data, with the stipulation that they would be used solely for scientific purposes. The study participants were assured of the anonymity of the study, which means that it is not possible to identify the individual being studied as a participant in the study/an individual with NF1. The possibility of identifying the study participants was maintained only by the researcher conducting the study and only for the purpose of analysing the collected data. The researcher informed the participants about the legal obligation imposed on her to protect the data collected during the interviews, as well as about the protection of this data by the Personal Data Administrator of Jan Długosz University. Based on this information, the individuals with NF1 decided to participate in the study. All the participants expressed their oral, informed, verbal consent to participate in the study, after which a convenient date for the interview was set in conditions ensuring the privacy of the respondent and the absence of interfering factors. The act of giving consent by the study participants was recorded in the audio recording of the interview and included in the transcript.

The study group consisted of 93 individuals. Each participant underwent an IDI, which, due to its interpretative potential, enabled a thorough understanding of the ways in which NF1 individuals experienced their disease, the meanings given to the disease, and being sick. The interviews were based on a general scenario containing open questions, and based on the respondents’ answers, the researcher explored specific themes using a semi-structured list of auxiliary questions. The part of the study devoted to experiencing NF1 in the reproductive dimension included questions on the following issues: (1) plans related to starting a family and having children; (2) assessment of the difficulty of the decision to become a parent and the dilemmas associated with it.

In order to obtain the most detailed, reliable, and truthful data, the researcher asked open and non-judgmental questions, focusing on the experiences of the respondents and the meanings they gave to the events and situations related to NF1 they described. The participants were encouraged to openly share their reflections, also on personal and intimate topics. The respondents were also informed about the possibility of withdrawing from the study at any time or refusing to disclose information about their personal situation without any consequences. Because some of the questions refer to sensitive issues that may cause emotional discomfort or stress resulting from considering difficult topics or past decisions, the interviewees were also instructed that they could ask for a break to decide whether to continue the interview. The interviews lasted approximately 90 min on average, and the statements were recorded using a dictaphone with the respondents’ consent. The participants were mostly in their own homes during the interviews, which contributed to their sense of comfort and privacy.

A total of 134 h, 26 min, and 35 s of audio material was collected and then transcribed in a naturalized form. None of the participants asked for authorization to transcribe the interview. Owing to the wealth of collected data, there was no need to conduct repeat interviews.

### Data analysis

Qualitative data analysis was conducted according to Kathy Charmaz’s [16] grounded theory methodology by the first author of the manuscript (KK). The process involved three stages of coding: (1) Line-by-line coding, which took the form of verb coding. (2) Focused coding, which enabled the extraction of the most analytically significant and frequently occurring codes. (3) Theoretical coding, which consisted of integrating the focused codes into conceptual categories that made the analyses more precise and coherent. Barney Glaser’s [17] “Six Cs” framework was used here.

The research was conducted within the framework of constructivist grounded theory, which involved avoiding preconceived assumptions and maintaining openness to new themes emerging during the analysis of the collected data. This attitude of the researcher is consistent with the procedure of grounded theory methodology, concerning maintaining the context of discovery (serendipity) [18]. The analysis of the empirical material continued until saturation was achieved, at which point the researcher determined that the developed analytical categories accurately captured the experiences of the study participants.

The theoretical sampling strategy was used in the research process, which allowed the selection of participants with different degrees of biosomatic manifestation of NF1 and comparison of their experiences of the disease in accordance with the constant comparative method. This activity lasted until theoretical saturation was achieved, i.e., the situation of data repetition and their analytical exhaustion.

## Results

Experiencing a body changed by NF1 and how the disease is lived with significantly impacts the decision to have children. While image-related issues play a role and can complicate functioning in intimate relationships, the most significant factor is the genetic and hereditary nature of NF1. This often leads many couples to decide against having children, which, alongside the challenges of building a lasting relationship and starting a family, is considered the most painful and overwhelming consequence of NF1. Both individuals who do not have children and those who choose not to have another child described this aspect as painful and destructive.

### Reproduction as an (un)ethical practice

In childless individuals, the decision to resign from having children primarily resulted from the awareness of the risk of passing on the gene mutation to the child. In turn, in families where the disease has a hereditary basis, this decision was to break the generational relay of NF1 inheritance. Notably, individuals from families with a confirmed positive family history expressed a sense of intergenerational responsibility more often, emphasizing that they did not want to continue the cycle of the disease. In contrast, participants with *de novo* mutations, although equally aware of the genetic risk, less frequently framed their decision as a response to familial legacy and more often emphasized individual ethical responsibility. While in both cases resigning from having children was an expression of the respondents’ rationality and parental responsibility, conceiving a child, despite the awareness of the genetic risk, was considered an unethical decision:

*I don’t plan on having children because of my knowledge*,* my medical knowledge*,* my biological knowledge. I think it would be simply unethical. I wouldn’t feel good knowing that I have a 50% chance of having a sick child. My child will have a 50% chance of inheriting the disease from me. And I think it’s so unethical*,* knowing about the risk*,* knowing what the disease may involve. As you know very well*,* you cannot even know that you are sick*,* because the gene expression is very low*,* but very serious deformities can occur. I simply think it would not be ethical that know about the risk if I were to decide to have a child.* [Interview 53]

*The most important aspect for me is that I would not put the child at risk. Because the probability that he will have this disease is very high*,* as I have an intense disease*,* so there is a high probability. The doctor said it’s 50/50 (…). And I know how it looks for me*,* so there is no way I would endanger a child. Because then it would be a conscious action in a sense. And it would be immoral on my part.* [Interview 47]

The common denominator of both statements is a strong sense of ethical responsibility. The respondents perceived the decision not to have children as morally right in the context of the risk of passing on a genetic disease (NF1). Awareness of the heredity of the disease (a 50% risk) and potentially severe symptoms encouraged them to consider procreative decisions as not only personal but also ethical choices. The foundation of these decisions is medical knowledge from a doctor. The study participants demonstrated a good knowledge of the mechanisms of inheritance and the course of NF1, which affects the way they think about parenthood. This knowledge not only increased their awareness of the risk but also emphasized the importance of consciously making reproductive decisions.

The deeply rooted fear of the child’s suffering was also strongly emphasized. The main reason for resigning from parenthood was the fear of exposing the future child to deformities and the possibility of developing serious health problems resulting from a severe course of NF1. The prospect of “consciously exposing” a child to NF1 is unequivocally assessed as immoral. The projection of the parent’s disease and the belief that it will be similar in the child also have a major impact on the decisions to refrain from procreation in individuals with NF1. The statements of the study participants indicate that these decisions were strongly rooted in their own personal experiences related to the disease.

### Reproductive dilemmas: not living versus experiencing NF1

Pointing to the social dimension of neurofibromatosis, the respondents emphasized that resigning from having children was supposed to protect the offspring from experiencing similar problems that the respondents themselves have experienced in their lives. The main concerns of the respondents were related to the stigmatization and isolation of the child. It was even suggested that not living is better than living with NF1 and its consequences. Using the medical model of the disease, it was emphasized that NF1 is a disease with an unpredictable, individualized course as well as uncertain prognosis, and its mild form in parents does not necessarily mean a mild form of the disease in the child:

*It would hurt me the most if a child was born with the same disease as I*,* because I would never want my child to go through what I have. I’d rather he didn’t exist than for him to be oppressed as much as I was oppressed. (…) And that would hurt me the most*,* the harm to my child.* [Interview 10]

*It goes like this with every line of kinship*,* that the course is worse. For example*,* I said my grandfather had NF1*,* but in my grandfather*,* there were only small subcutaneous nodules. In my case*,* they are already in the skin and on the muscles. At the moment*,* I do not want to have children*,* and this is only related to the disease.* [Interview 54]

*I don’t want to have children. If I have a child who is going to have NF1*,* I can’t imagine going through what my parents went through*,* so no*,* no*,* no. And it’s like fifty-fifty*,* so I don’t want to. At the moment*,* I definitely wouldn’t want a child with the same disease as me. Because what I went through… I don’t want it to happen again*,* I don’t want to look at it. My story would come back to me*,* and I would have to go through it all over again*,* and probably even worse*,* because if it was my child*,* I would be even more upset*,* even more stressed*,* and if he had even worse symptoms*,* I wouldn’t be able to cope. I think I definitely wouldn’t be able to cope with it.* [Interview 64]

The interviewees’ narratives mentioned above are filled with a strong sense of empathy and identification with the future child. The study participants project their own, often traumatic experiences onto the imagined life of the child. The suffering of the offspring would be more painful for them than their own – there is a strong fear of a “repeat” of traumas, this time experienced from the position of a parent. Parenthood means for them the necessity to relive their own experiences, which is too burdensome. Resigning from parenthood, therefore, appears as a form of emotional insurance. The participants declare that they would not be able to emotionally face the situation if the child were sick with NF1. These fears are additionally intensified by their own conviction that the NF1 symptoms will intensify in subsequent generations. This additionally strengthens the concerns related to the possibility of deterioration in the child’s quality of life and confirms the motivation to withdraw from parenthood.

### Taking care of one’s own health

Women whose doctor predicted a difficult pregnancy because of NF1 or who feared the consequences of pregnancy on their own disease course justified their decision to resign from having children with concern for their own health. While pointing out the potential negative consequences of pregnancy, the right to care for one’s own health was also emphasized.

*Should I have children or not? I think that for me*,* pregnancy is a threat to my life. Because a tumour can grow*,* new tumours can appear*,* and the ones that are there can also start to grow. Spinal curvature*,* because I have a big deformity here. And pregnancy is a personal threat to my life. And I know that I have the right to take care of myself*,* and no one can force me to have a child because it’s appropriate*,* because it’s necessary*,* or whatever.* [Interview 1]

*The only thing I can do is not accelerate the disease with hormones. I am giving up on having children because I am afraid of what these pregnancy hormones can do to me*. [Interview 2]

*I will not decide to have another child because I barely survived the first pregnancy. I had to undergo a heart procedure while pregnant*,* which was done using an innovative method*,* without the use of X-rays. After this first pregnancy*,* I am afraid to decide to have another child because I don’t know if I would survive another pregnancy. I would not like to experience the same thing twice*! [Interview 43]

A common question that appeared in the collected empirical material was the existential question: “Should I decide to have children at all?”. This question was not always asked in the context of caring for the child. It was also based on the risk of health complications in the mother, sometimes confirmed by past experiences. Pregnancy was perceived by women with NF1 as a real threat to their health and even life. The study participants were afraid of their own health deterioration during pregnancy. They were also afraid that pregnancy could worsen the symptoms of NF1 (e.g., bone deformities) or even lead to serious complications. In particular, they were afraid that the hormonal changes accompanying pregnancy could stimulate the development of skin lesions (an increase in the number of tumours). For these women, giving up motherhood becomes a strategy to “not accelerate” the progression of the disease.

Another factor in the decision-making process regarding motherhood was the traumatic experience of previous pregnancies. The decision not to have another child results directly from the extreme complications of the previous pregnancy. This experience left a lasting emotional mark and a strong fear of exposing oneself to danger again. The narratives of the respondents emphasized the right to decide about one’s body and health. The above statements also contain elements of resistance to social norms and pressures regarding motherhood. The study participants clearly articulated their right to resign from parenthood as an act of self-care.

### The social construction of genetic risk

Furthermore, the individuals who declared they would not have another child emphasized that this was due to the risk of passing on a gene mutation to the child. And although this fear was present regardless of the health status of the child/children, the presence of NF1 in the child was a factor that strongly inhibited the procreation plans of the respondents, especially in the face of the risk of a more severe course of the disease than in the sick parent.

*So*,* no*,* I don’t plan on having children. I don’t plan on having them today. Well*,* that’s too much of a risk. Because it’s 50%*,* 50/50. Just because [child’s name] has such mild symptoms doesn’t mean my next child might have the same. I’d be scared. I wouldn’t consciously decide to have another child*. [Interview 40]

*At the genetic clinic*,* I learned that if no spot or lump appears in him [son] by the age of 3*,* it means he is healthy. However*,* those first three years were terrible. I observed him even more and saw something in him that I had as a child (…). Whenever I saw something*,* some disorder*,* that something was wrong*,* I was afraid of this disease. Fortunately*,* he is healthy*,* and everything is fine. But I was afraid to decide to have a second child*,* because I was really afraid that NF1 might become activated in the second one.* [Interview 77]

The unpredictability of the course of the disease was a source of fear before deciding to have another child. The phenotypic variability of NF1 significantly complicated procreative decisions, which were associated with great stress and uncertainty in the women studied. The experience of waiting for a diagnosis in a child also occurred to be traumatic in the experiences of mothers. Careful, even neurotic observation of the child’s body during the first years of its life, searching for symptoms and the constant fear of confirming NF1, made it impossible to take a conscious decision to have another child.

Nevertheless, the key factor in the decision to resign from parenthood remains the 50% genetic risk, which the subjects were unable to ignore even when they already had a healthy child. The fact of having a healthy child did not free them from the fear that the next child would be born sick. By emphasizing the high level of risk and describing previous experiences in terms of luck, the subjects were convinced that they had “exhausted the prize pool in the genetic roulette”.

*I gave birth to a healthy child*,* and I think I have used up my luck. I didn’t realize*,* even though I knew it would be a high-risk pregnancy*,* that I didn’t realize the effort my doctor would make to carry out this pregnancy*,* and I think I shouldn’t tempt fate. (…) The fear that this time I will give birth to a sick child…* [Interview 51].

*So I did everything with full awareness. From the beginning*,* I planned that the child had to be healthy. It may sound strange*,* but I didn’t even accept the information that the child would be sick. Of course*,* there was some fear*,* because*,* of course*,* I was worried about the whole pregnancy*,* but I was rather focused on the fact that the child would take on my husband’s genes and everything would be fine. And that’s what happened. My son is 12 years old. He’s healthy. Well*,* I think nothing can happen now. He got the good genes. But we’re not planning on having more children; we’re afraid of this disease.* [Interview 56]

The decision to resign from further parenthood was also influenced by a previous pregnancy, which the respondents presented as a demanding medical experience. One of the respondents drew attention to the enormous effort doctors need to make to maintain the pregnancy, which additionally strengthens the belief in its particular risk.

The conscious nature of the respondents’ decision to become pregnant was emphasized here, which, nonetheless, did not free them from genetic anxiety. As a coping strategy, one of the women used conscious control of the narrative about the child’s health. She developed a kind of mechanism of “programming” herself to give birth to a healthy child. The woman rejected thoughts about the possibility of the child’s disease, which was her way of dealing with the stress of pregnancy. Even though the woman gave birth to a healthy son, the fear of NF1 remains so strong that the decision to have another child was rejected. The genetic risk of NF1 remained a dominant, blocking factor in planning further offspring.

### The right to have children

Some of the study participants declared a constant desire to have children, despite being fully aware of their own disease and the risk of passing on the gene mutation to their offspring. Their interpretations also included fears of the child inheriting NF1 and its potential suffering. However, this group of respondents emphasized more strongly the autonomy and individual right to be a parent, despite the disease. The decision about their potential parenthood was not blocked by the awareness of the genetic risk. The strong motivation to be a parent, the desire for a family, and personal values ​​outweighed the fear related to the disease.

*Yes*,* I am planning a child and I am desperate*,* because I am 41 this year and I told my husband that I want to become a mother for the second time this year*,* very much. We will see if nature allows me to do it*,* if my disease allows me to have another child*,* because I would like it very much. My husband already stated during my first pregnancy that whatever happens*,* he will be with me 100%. Whatever decision I make at the beginning of the pregnancy*,* he will be with me. But there was nothing in me that I would not be a mother because of this disease. We did not think that way. We wanted to have a family despite the disease. (…) I really stopped looking at myself through the prism that I am sick*,* that I have a tumour here. Even though I had a hard time with my first pregnancy*,* I will decide again. (…) I knew that a mother passes on this disease in 50%. Today*,* my son does not have the disease*,* but he is only 6 years old*,* and I am still terrified of what will happen in 2–3 years*,* and there is still a great unknown. But I wanted to be a mother from the beginning; my husband and I really wanted to be parents.* [Interview 79]

*When I was single*,* I was ready to revise my plans and give up on having children*,* but I didn’t give up because the person I’m with now*,* the person I’m dating*,* just like me*,* accepts this risk*,* even if the child were born with this disease*,* he simply says: “Too bad*,* you’ll love him even more”. You know*,* I met someone for whom my disease is not a problem and is not an obstacle that closes everything.* [Interview 8].

The key role in deciding parenthood was played by the support of a close person. Acceptance and understanding from a partner, also in the face of potential difficulties related to the child’s disease, gave the respondents a sense of security and space to make decisions in line with their desires. Individuals with NF1 who were not in a relationship focused on finding the “right” person, i.e., someone who would confirm their decision to procreate.

The decision about motherhood/fatherhood was also made taking into account the available methods of reducing the risk. The respondents indicated several strategies to facilitate their procreative decisions, including conceiving a child using in vitro fertilization with preimplantation genetic diagnostics, sperm selection tests, or the genetic testing of egg cells. Wanting to increase the chances of having a healthy child, they actively sought solutions that would allow them to take control of the risk.

*I definitely don’t want to get pregnant naturally*,* but by in vitro. I know that it is possible to test the eggs so that they are not burdened with this defect. I want to make a conscious decision about having children*,* because I simply don’t want my children to inherit this disease from me. And apart from that*,* I plan to live normally and try to live*,* function normally. To be as happy as possible.* [Interview 39]

*Of course*,* I plan to have children. Of course I do. But I’m also scared. That’s why I’m going to do a sperm segregation test. I know that in this case it’s possible. Because I wouldn’t want a sick child*,* since it would involve a lot of sacrifices*,* and I wouldn’t want the child to suffer. I want to have a healthy son*,* and that’s it. We both want to have a son. So we’re planning a child*,* absolutely.* [Interview 63]

To sum up, the above statements are filled with fear but also determination. Although the respondents declared their desire to have children, it was accompanied by uncertainty – especially in the face of the unpredictable course of NF1. These fears did not block action, but were part of the process of making conscious, well-considered procreative decisions.

### The desire to have children

The decision to have children was made mainly by individuals who experience a mild course of the disease. Emphasizing that they did not feel sick, they were ready to take the risk associated with the potential disease of their child. For them, NF1 was therefore only a medical diagnosis, not a real reality that changes the way they function every day. Not experiencing limitations related to the manifestation of disease symptoms, including those related to body image and aesthetics, they did not define themselves within the framework of a disease identity. These cases often included individuals with a mild phenotype or uncertain family history, which allowed greater openness toward parenthood despite known risks. A mild course of the disease meant that for some couples, NF1 is an unrealistic experience that does not provide grounds for limiting reproductive plans. On the other hand, the awareness of the risk of inheriting the disease by the child meant that these respondents did not define themselves as fully healthy individuals either. The identity of this group of respondents was an identity “between health and disease”, which may result in a temporary suspension of reproductive plans that may be significantly influenced by therapeutic relationships dominated by the discourse of risk.

*We wanted to have another one*,* but the doctor told my wife so much that we didn’t want to; we were simply afraid of this disease. Because Mrs. [name of doctor] said that it could be 50/50. It could be nothing*,* or it could be worse*,* or it could be the same. It could be different. She made us understand not to. She advised us against having another child. (…) We would like to have another child. If the disease was at the same stage*,* it would be no problem*,* because nothing like that happens. There’s nothing. (…) My daughter is sick*,* but there is no problem; there is absolutely nothing wrong with anything. We only have to do these tests once a year*,* go to the doctor*,* to just check if everything is OK*,* and they invite her back next year.* [Interview 20]

The decision not to have another child was not based on personal beliefs or experiences in the case of the interviewee, but was largely shaped by the opinion of a medical professionals. In this case, it was the doctor’s suggestion and the way she presented the genetic risk that influenced the change in the procreative decision, despite the previous desire to enlarge the family and the experience with the first child, whose disease was mild. The doctor directly advised against having another child, which, together with the presentation of its possible effects, contributed to an increased sense of fear and uncertainty in the sick parent. Medical recommendations, especially those formulated unambiguously or suggestively, can be discouraging and outweigh the individual’s sense of readiness for parenthood.

### Genetic culpability

Some of the respondents experiencing a mild course of NF1, who were parents of children with a different course of the disease, became parents being unaware of their own condition. These respondents learned of the NF1 diagnosis only when their child was diagnosed with the disease or when symptoms similar to neurofibromatosis appeared. The statements of these parents were full of sadness, regret, and fear.

*When I was 44*,* because my son was born in 2004*,* tests were done for it right away. And thanks to my son*,* I found out that my son has it and I have it. It was sad. A person has such a thing*,* and no one knew about it. I am most worried about my son. He has these lumps practically everywhere.* [Interview 31]

*A son was born*,* and the son is sick. He was immediately diagnosed as a sick person*,* as a person who has neurofibromatosis. Genetic tests were done. I mean*,* they didn’t really have to be*,* because once the doctor looked at me and at him*,* well*,* they knew that this was definitely*,* definitely going to be it. So then it came out*,* then I found out that it wasn’t just my nature*,* but that it was a disease*,* that it was a serious disease.* [Interview 86]

Thus, for some of the respondents, the diagnosis of NF1 in a child became a turning point for the parent as well, both emotionally and cognitively. It was only the birth of a sick child and the related diagnostics that revealed the presence of the disease in the parent, previously undiagnosed or downplayed (“That’s just the way it is”). In the above accounts, concern for the child and the burden of stress resulting from observing more severe symptoms in the child than in oneself were particularly resonant. The diagnosis, therefore, became not only a turning point in one’s own health awareness but also a source of fear for the child’s future.

Some of these parents also felt a strong sense of guilt related to the lack of knowledge about the risk and passing on the disease to their child. Emphasizing the empowering nature of genetic knowledge, the respondents indicated that earlier knowledge about NF1 could have had an impact on their reproductive decisions.

*The moment I found out that my son had it and I had it too*,* I was just really scared. I just couldn’t understand it. I said*,* “Why me*,* why my son?”. I didn’t know anything about the disease before I got pregnant. If I had known*,* I probably wouldn’t have decided to have a child*. [Interview 32]

*Well*,* maybe I regret that I found out about it so late*,* because a person wouldn’t decide to have children. I would prefer to be aware that I was sick earlier. I would have liked to have known. To have that choice. And not that I found out about it through my daughter*,* because of her disease.* [Interview 93]

These statements show the importance of disease awareness as a condition for making well-considered reproductive decisions. The respondents who learned about their NF1 diagnosis only after their child was born expressed regret about not being able to make an individual choice about whether to become a parent in the face of an incurable, rare genetic disease. Instead, they had to face a sense of losing control over their reproductive life. Some openly declared that they would not have decided to have a child if they had known about their own disease. This indicates the important role of access to information and genetic diagnosis in the family planning process. We should also not forget about the emotional consequences of a late diagnosis, which include fear, surprise, and difficulty in coming to terms with the information about both their own and their child’s disease.

### To break the spiral of inherence

The respondents with a mild course of NF1 emphasized at the same time that the fact that neurofibromatosis occurred in their first child and that they learned about their own medical diagnosis significantly limited their further reproductive plans. For some parents, the hereditary nature of NF1 raised concerns about the child’s justified, in their opinion, claims that the parents consciously transmitted the disease. For others, such reactions were a real experience of the parental relationship:

*Well*,* my wife and I would definitely like to have a child*,* but there is this fear now that it is 50/50. The longer I am sick*,* the more I wonder… Right now*,* I have such a conscious choice. Wouldn’t the child have a grudge against me later? That you knew*,* and yet somehow you brought me into this world. Because here*,* when it comes to my son*,* I had no influence on it*,* I didn’t even know that I had something like that*,* and that I passed it on to him. It wasn’t my doing; I didn’t know then. And here*,* though*,* I would have it*. [Interview 23]

*My daughter has emotional overexcitability*,* so reactions vary. But when it comes to my disease*,* sometimes in anger*,* she would ask me why I gave birth to her*,* because she was sick. It seems to me that it is mainly in anger*,* because she also feels that she has some limitations*. [Interview 28]

The quoted statements strongly emphasize the fear of being judged by the child and the moral burden of the decision to procreate made with full awareness of the genetic risk. The respondents asked themselves questions about the ethics of consciously passing on the disease, and also expressed concerns about the emotional, moral, and relational consequences of this decision in the future, including the possibility of experiencing reproaches from their offspring. These dilemmas became more intense with the lengthening of the time of experiencing one’s own disease. There was also a reflection on the difference between ignorance and awareness of NF1. The decision to have a child in conditions of ignorance was devoid of guilt, while the situation in which the parent knows the risk creates a much greater moral and psychological burden. These statements indicate an internal conflict between the desire to have a child and the responsibility, as well as concern for the future relationship with the child.

### Keeping the disease away from reproductive plans

Individuals with severe neurofibromatosis decided to have children despite medical contraindications, awareness, and concerns related to the risk of the disease in the child. By not capitulating to the disease, keeping it away from their personal identity, these respondents claimed that NF1 could not deprive them of the right to have the desired child. Considering their experiences in coping with their own disease, they indicated that the risk of inheriting the disease itself was not something they would not be able to cope with. The disease was something they knew well, were familiar with, which is why they were not afraid of realizing their non-disease identities as a mother or father. Therefore, this group also included individuals who planned or had already realized plans to have children, considering parenthood to be an important element in creating a non-disease identity.

*In my case*,* the disease is more acute*,* because today I have more and more tumours*,* they bother me more and more. (…) I plan to have children. I want to have another child. I would really like to have another child! I want to get pregnant this year. (…) Because a child really changes a lot. I don’t know if it’s my case or other women who suffer from NF1*,* but a child really brightened me up. I really stopped*,* as I said before*,* I stopped looking at myself through the prism of*,* and because I’m sick*,* and because I have one more or one less tumour here*,* but a child really changes a lot.* [Interview 79]

*I mean*,* I’ve lived with this disease my whole life*,* and nothing has happened to me*,* and nothing is happening to me. (…) Although I had a head MRI once and nothing came up. I don’t worry about my disease. I haven’t really felt sick since I had kids. My son has a very severe form*,* my daughter less so.* [Interview 3]

The respondents emphasized the transformative power of the experience of parenthood, which, in their opinion, brings emotional relief, a change of perspective, and a sense of meaning, despite the disease. The child becomes a source of motivation, brings a sense of normality, and even “brightens up” life. Some of the respondents also showed an attitude of trivializing their own health condition in the context of the more serious symptoms of the child’s disease, which may indicate a strong identification with the role of caregiver and a greater focus on the needs of the offspring than their own.

## Discussion

A genetic disease affects every dimension of an individual’s life and transforms how they perceive and experience their body and identity, which are framed in genetic terms. It also transforms family relations, which become more risky. Nevertheless, it is reproduction and family planning that seem to be the most disrupted, as both pregnancy and parenthood become increasingly understood and experienced as tentative and morally problematic [19–23]. While the decision to have a child always involves emotional and psychological considerations, individuals affected by a rare hereditary disease experience prolonged anxiety over the potential effect of their condition on pregnancy and the likelihood that a child may inherit the “bad” gene [23, 24]. This finding is consistent with studies on other hereditary cancer syndromes, such as BRCA1/2, Li-Fraumeni Syndrome, and Lynch Syndrome, which demonstrate that individuals often perceive a strong sense of genetic responsibility and moral obligation to prevent transmission of the mutation to their children [25–27]. These conditions similarly evoke tension between reproductive desire and risk management. Hence, by showing that individuals with NF1 frequently have to make tough reproductive decisions when planning their families [13, 28–30], our study yielded several important findings.

Firstly, it shows that resigning from having children is one of the most overwhelming losses experienced by individuals with NF1. Simultaneously, although such a decision was often influenced by the physical changes caused by the disease in one’s body, and the disruption it causes in intimate relationships with the partner, this study confirms previous findings showing that in the case of hereditary diseases, such as NF1, the awareness of the genetic risk is the single most important factor [13, 28–30]. In BRCA mutation carriers, awareness of reproductive options such as preimplantation genetic testing (PGT) is relatively high, but uptake remains limited, suggesting a complex interplay between knowledge, perceived disease severity, and personal values [27, 31]. Nonetheless, this should not surprise them, as “new genetics” focuses on both knowing and managing risk, and genetic information becomes an essential component of the reproductive process, preconception, and prenatal care, in addition to disease prevention [19, 20, 32].

Interestingly, our data suggest that the perception of reproductive responsibility may be modulated by the origin of the mutation: participants with *de novo* mutations more frequently described their decision-making as an isolated moral choice, whereas those with inherited NF1 often referred to the family history of suffering and the desire to end the inheritance of the disease. This resonates with findings by Dimond [33], who showed that individuals with *de novo* mutations often frame reproductive decisions as highly personal and ethically charged, due to the absence of shared family experience or history, and the resulting burden of individual responsibility. Similarly, Onnekink et al. [34] demonstrated that in families with CDKN2A mutations, a positive family history of cancer strongly shaped reproductive decisions; participants with affected relatives were more likely to reference inherited suffering and the intergenerational desire to “end the chain”, whereas those without such a history tended to frame decisions in more abstract or individualistic terms.

Moreover, although NF1 severity was not a formal stratification factor in our analysis, qualitative differences emerged: participants with moderate to severe NF1 more often emphasized bodily limitations, stigmatization, and the unpredictability of disease progression as key reasons for avoiding parenthood. Conversely, those with mild symptoms tended to express ambivalence, sometimes leaving the decision open or conditional on future health status. These findings confirm previous research showing that reproductive choices in hereditary conditions are shaped not only by statistical risk but also by lived embodiment, emotional memory, and perceived social visibility of the disease [33, 34].

Secondly, since genetic information about an individual applies to others and is perceived as a particular type of health risk information, this study confirms that the management of genetic risk is framed as a familial obligation [22, 35]. In fact, although approximately 30%-50% of NF1 mutations occur *de novo*, most parents are aware that, as a consequence of the autosomal dominant inheritance pattern, the mutated gene is 50% likely to be present in their future children. In contrast to individuals with recessive or X-linked conditions, NF1 patients, similar to those with dominant conditions like Li-Fraumeni Syndrome, frame this 50% risk as near certainty, which strongly shapes their reproductive choices [26, 36]. A recent study of CDKN2A mutation carriers also confirms that although many are aware of their genetic risk, few consider PGT, pointing to persistent gaps between knowledge, emotional readiness, and practical accessibility [34]. Thus, even though in the case of NF1 the genetic risk entails a statistically defined probability of a disease, many respondents framed it in terms of certainty rather than chance [37]. This confirms with previous studies showing that even though many individuals with NF1 are aware of the probabilistic nature of the risk of passing on a gene mutation to their child, they still believe it is too high [13, 28–30]. It also confirms that while individuals’ reproductive choices are influenced by a variety of variables, including their own experiences with the condition, its severity (both in terms of physical symptoms and social stigmatization), worries about the effects of pregnancy on their health and body, it was the hereditary risk of NF1 that was the most important factor influencing the respondents’ decision about not having (subsequent) children [23, 28–30]. This is consistent with our findings, where respondents with more severe symptoms expressed greater reluctance to have children, while those with milder forms of NF1 or uncertain family histories were more likely to consider parenthood [33, 34].

Thirdly, while highlighting the importance of risk perception for reproductive decision-making, this study emphasizes that most respondents emphasized the internal and individual character of genetic risk, as well as internalized the imperative of knowing and managing the risk by making “rational” choices [22, 24, 37]. Thus, it confirms that as genetic discourse becomes highly individualized, there is also a tendency to its moralization [20]. By referring to the sense of genetic responsibility, both individuals who had children and those who had not stressed that, as a (prospective) parent, one must ensure the health of the future child. This supports broader trends in genetic counseling and ethics, where individuals increasingly frame reproduction through moral obligations to reduce harm, especially in hereditary cancer syndromes like BRCA and VHL [38, 39]. It was also suggested that deliberately passing on “faulty” genes and exposing a child to disease, which can be prevented, is selfish, irrational, and morally wrong [26, 40]. Consequently, the decision to refrain from having children was perceived by many as a sign of (genetic) prudence, rationality, in addition to responsibility for others, and was framed as a protective reproductive practice that prevents children from suffering [20, 22]. Indeed, studies of BRCA1/2 mutation carriers show that refraining from childbearing can be seen as a rational and altruistic act, despite advances in genetic screening options [41]. Thus, this study confirms that modern genetics strengthens the conviction that individuals should be held morally accountable for conceiving a child who will have the “best chances of the best life” and preventing the “harm of existence” [42]. In contrast, genetic ignorance or the desire to have children despite the risks of NF1 was seen as an expression of selfishness, irrationality, and irresponsibility that jeopardizes children’s health [26, 43].

Previous studies also demonstrated that while many adults with NF1 exhibit a wish to have disease-free children, they are anxious about the possibility of passing NF1 on to their children, possible childbirth treatments, and doubts about finding a partner [28, 29]. Consequently, they often stressed their duty to ensure the child’s welfare; they stressed that it is immoral to expose a child to the risk of NF1 and “sentence” them to suffering, resulting not only from the uncertain and progressive nature of the disease but also its social consequences [23, 28–30]. Similar psychosocial concerns were observed in individuals with hereditary breast and ovarian cancer, who feared stigmatization and viewed transmission of the mutation as morally and socially consequential [38]. Moreover, since even individuals with mild symptoms of NF1 stressed the unpredictable disease progression, which may result in the fact that their children may experience more severe symptoms than their parents [28], this study confirms that it is “quality of life” that is the primary focus of many parents, who tend to suggest that not living is better than living with a disease [42]. Significantly, both in individuals who already had healthy children and those who were childless, the perception of genetic risk was stronger than their tentative desire to have (more) children. Thus, irrespective of having (healthy) children, individuals with NF1 often suggest “reaching their luck limit”.

Fourthly, although fertility in individuals with NF1 is not particularly affected by the disease, women in particular were concerned that pregnancy could jeopardise their health and well-being. This supports previous findings showing that women with NF1 have a higher-than-expected risk of spontaneous miscarriages in the first trimester, premature delivery, and an increased rate of caesarean section, intrauterine growth retardation, and stillbirth. Pregnant women with NF1 are also more susceptible to hypertension and cerebrovascular problems [3, 44–46]. Moreover, like many other skin diseases, NF1 significantly affects how individuals feel about their bodies, their sexuality, and themselves. A recent study reported that individuals with NF1 have a more negative body image in addition to worse sexual self-esteem and report a lower quality of life than healthy individuals [47].

Fifthly, since some individuals with NF1 live in the shadow of their disease, this study shows that the discourse of genetic responsibility is associated with a discourse of guilt and blame [33]. In fact, some parents experienced feelings of guilt about not recognizing the risk and passing on the disease to their children, and suggested that prior knowledge of NF1 could have influenced their reproductive choices. However, previous studies have also reported increased maternal guilt and blame resulting from X-linked inheritance patterns, such as in Duchenne/Becker muscular dystrophy and spinal muscular atrophy types II/III [48–50], as well as among female BRCA1/2 mutation carriers and in conditions like Von Hippel-Lindau and Li-Fraumeni syndromes [31, 36, 39].

Lastly, although all the respondents were aware of the risk of passing on the “faulty genes” to their children, and emphasized how the hereditary of NF1 interfered with their ability to have children, some individuals reject the discourse of genetic responsibility by referring to one’s desire to have children, which was defined as a basic human right [51]. Thus, since children were perceived as the most significant element of the family, which, in turn, is considered the most essential value in Polish culture, some individuals with NF1 argued that the discourse of genetic risk endangers an individual’s autonomy and right to an open future. Still, some individuals with hereditary cancer syndromes reject the deterministic logic of genetics and emphasize autonomy and the right to family life, framing children as a source of personal identity and healing [26, 38]. Hence, this study shows that although genetic disease significantly shapes self-image, and many individuals define themselves in genetic terms, especially those with mild symptoms or without children, reject the discourse of genetic fatalism [52] and NF1 as a self-identity and “master status” that encompasses all other traits of the individual. By rejecting genetic stigma, they emphasized that having a child gives them a sense of normal life, and by stressing chance and luck instead of risk, suggested that the moralization of genetic discourse cannot deprive them of their right to have their desired child. Nevertheless, it was demonstrated that such decisions are not only an expression of objection against the moral dimension of genetic discourse but also a parental strategy to reject the imperative of knowledge [51, 53].

## Limitations

While this is one of the few studies on reproductive choices and family planning in individuals with NF1 in Poland, it has some limitations. Firstly, although 93 respondents were interviewed, the actual number of individuals with NF1 in the country is certainly higher. Therefore, the findings cannot be generalized for the entire population of individuals with NF1 in Poland; rather, they reflect the views of the patients who consented to participate in the study. Nonetheless, it should be acknowledged that because Poland still lacks a register of rare diseases, the exact number of individuals with NF1 in the country is unknown. Secondly, owing to the qualitative design of this study, the results cannot be broadly applied. Thirdly, since this study has a local focus, comparing these findings with those from other countries would be desirable. Fourthly, since the study participants were recruited with the help of two doctors from one hospital only, some individuals with NF1 from other medical centres or those who did not have contact with these doctors could have been omitted. Fifthly, although the respondents experienced various stages of the disease, relatively few experienced advanced stages of NF1. Meanwhile, the experiences of individuals with an advanced stage of the disease may vary. In addition, although our sample included participants with varying NF1 severity and different inheritance patterns (including both *de novo* and familial cases), the study was not designed to systematically analyse correlations between these clinical variables and reproductive decision-making. Future research should further explore how confirmed mutation status, inheritance pattern, and disease severity interact with psychosocial and ethical dimensions of reproductive choices. Sixthly, most of the respondents were females, which limits the transferability of the results to male participants. Seventh, the study was conducted in the form of telephone or online interviews, which made it difficult to observe the behaviour, gestures, and emotional states of the participants. Thus, while this may have encouraged greater openness on the part of the respondents, it made it difficult to contextualize the interviewees by observing non-verbal messages that are possible to grasp in a face-to-face scenario. However, it should be emphasized that this limitation results from objective reasons, as the data were collected during the 2020 COVID-19 pandemic. Eighthly, because the data was collected, coded, and analysed by a single author, there was a risk of subjectivity affecting the selection of the topics and the interpretation of the data. Ninthly, although preimplantation genetic diagnosis (PGD) and other assisted reproductive technologies constitute an important dimension of reproductive decision-making among individuals with genetic risk, and several participants spontaneously mentioned these methods, the topic was not directly explored in the interview guide. As a result, data on the awareness, accessibility, and perceived barriers related to PGD among individuals with NF1 remain limited and require further investigation. Lastly, due to the sensitive nature of the study, some participants may have felt uncomfortable discussing the topics.

Despite these limitations, it is important to highlight the significant advantages of this study. Most importantly, it is the largest study conducted on the Polish NF1 community and the first to examine the reproductive dilemmas faced by individuals with NF1. Thus, it fills a gap in the literature on patients’ challenges and needs. Moreover, by identifying the main challenges and dilemmas, this study sheds new light on the impact of the disease on the quality of reproductive health in individuals with NF1. The study may, therefore, stimulate further research on the sexual and reproductive health of patients with rare genetic diseases. It may also help to identify actions that should be taken to support patients’ needs. Finally, since this study enabled the respondents to share their lived experiences, it might have a therapeutic value.

## Conclusions

Based on the presented results, the following conclusions can be drawn.

1. The awareness of genetic risk plays a dominant role in reproductive decision-making. The respondents, regardless of whether they were already parents or not, were aware of the mechanisms of NF1 inheritance and the 50% risk of passing on the disease to offspring. This knowledge had a significant impact on the decisions to resign from parenthood. The genetic risk of NF1, therefore, appears as a socially constructed decision-making factor. It is not only medical knowledge, but also becomes a prism through which the respondents assess their right to parenthood.
2. Resigning from parenthood is seen as an act of responsibility and moral concern. Knowingly exposing offspring to the suffering associated with the disease was described as unethical. The respondents experienced an internal conflict between the desire for parenthood and the ethical obligations resulting from medical knowledge.
3. The procreative decisions of individuals with NF1 are strongly rooted in personal experiences of the disease, equally physical, mental, and social. Experiencing the deformations and limitations of the body changed by the disease, mental suffering as a consequence of stigmatization and exclusion, in addition to experienced traumas, shaped negative ideas about the future of a potential offspring.
4. Pregnancy in women with NF1 is perceived as a threat to one’s health and life. The studied women were aware that becoming pregnant could accelerate and exacerbate the progression of the disease, and even cause life-threatening crisis episodes. Resigning from motherhood was therefore a strategy to protect one’s own health and life, while at the same time emphasizing their opposition to the social pressure to become mothers.
5. Motherhood for women with NF1 is experienced through the prism of severe stress and fear for the child. Waiting several years for the medical diagnosis of NF1 to be confirmed or ruled out was associated with experiencing chronic stress and uncertainty. Fear related to the inability to predict the severity of the disease and its impact on the child’s quality of life discouraged the respondents from planning further offspring, even if the first child was born healthy.
6. There are also voices affirming the right to parenthood despite NF1. Among the study participants were individuals who, despite being aware of the risk and their own condition, had decided or were planning to have children. The narrative of the right to a family and parental love dominated among these individuals, while accepting the potential health consequences for the child. Their decisions were motivated by loneliness, the need for closeness, or a strong desire to fulfil the role of a parent.
7. A late diagnosis of NF1, often occurring simultaneously with the diagnosis of one’s child, often becomes a source of guilt and a sense of loss of control over one’s reproductive life. The respondents indicated that an earlier diagnosis might have significantly affected their reproductive decisions. The child’s genetic burden is experienced as a moral burden by the parent.
8. Awareness of genetic risk often limits the willingness to continue parenting. Respondents expressed concern that their child might one day blame them for knowingly passing on the condition. Awareness of the disease transforms the nature of moral responsibility – from unconscious to deeply consequential, which significantly increases the psychological burden on the parent. Notably, the social dimension of the disease (fears of stigmatization, discrimination, and social isolation) often appears more pressing than its biological implications.
9. Parenthood for some individuals with NF1 plays an important role in redefining identity. Despite the serious course of NF1, some of the respondents decided to become parents, emphasizing its transformative and therapeutic nature. Here, the child is not only an object of care but also a symbol of normality and social embeddedness. In this way, the disease is “separated” from personal identity as it no longer fully defines the individual because a new social role appears – that of a mother or father.

The application value of the presented research and analyses prompts us to formulate several proposals for practical solutions in the field of the reproductive health of individuals with NF1, or more precisely, medical practice, mental support, and health policy.

1. Early genetic diagnosis is crucial, especially in families with a history of NF1. In addition to improving access to genetic testing, screening programs and training for primary care physicians should be introduced to detect the disease early in individuals with a first-degree relative with NF1, even if they do not experience severe symptoms.
2. Genetic counselling for individuals with NF1 should be developed. Couples planning a child should have the opportunity to participate in systematic genetic counselling so that they can make an informed decision about procreation based on full information about the risk of transmitting the disease to their offspring.
3. Couples in which one person has NF1 should gain better access to assisted reproductive technologies. Preimplantation diagnostics give a chance to make a more informed procreative decision.
4. Coordinated care for individuals with NF1 should include psychological support in the context of reproductive decisions and choices, especially dedicated to two groups. The first is comprised of those who learn about their neurofibromatosis at the time of diagnosis in their child. This support should be focused on working with the parents’ sense of guilt and fear for the child’s future. The second is composed of those who plan to have children. Psychological preparation for parenthood in the case of NF1 should be based on support programs for future parents that would take into account the emotional challenges related to reproductive decisions.
5. The activity of patient associations and self-help groups of individuals with NF1 should be strengthened in the area of ​​popularizing strategies for adapting to life with neurofibromatosis. It is important to strengthen attitudes towards NF1 in which, regardless of the problems associated with the disease, individuals with the disease do not feel completely defined by the disease and are able to fulfil their parental roles in a satisfactory way.

## Data Availability

Due to sensitivity concerns, the data underlying the results of this study are not publicly available; nevertheless, they can be obtained from the corresponding author upon reasonable request.

## Abbreviations

NF1: Neurofibromatosis type 1
